# Cerebral Ischemic Complications After Surgical Revascularization for Moyamoya Disease: Risk Factors and Development of a Predictive Model Based on Preoperative Nutritional Blood Parameters

**DOI:** 10.3389/fnut.2022.842838

**Published:** 2022-03-10

**Authors:** Fangbao Li, Chuanfeng Li, Yunwei Sun, Yue Bao, Wenbo Jiang, Zuoyan Song, Yongyi Wang, Mingxing Liu, Weimin Wang, Tong Li, Luo Li

**Affiliations:** ^1^Department of Neurosurgery, Qingdao Municipal Hospital, Qingdao, China; ^2^School of Medicine, Dalian Medical University, Dalian, China; ^3^Department of Anesthesiology, Qingdao Municipal Hospital, Qingdao, China

**Keywords:** moyamoya disease, postoperative cerebral ischemia, risk factor, nutritional parameters, predictive model

## Abstract

**Objectives:**

Cerebral ischemic complications are common after revascularization in patients with moyamoya disease (MMD). Risk factors from specific laboratory variables have only been assessed by limited research. This study was to investigate the association between postoperative cerebral ischemia and nutritional blood parameters and examine predictive values of such risk factors in adults.

**Methods:**

Preoperative demographics and nutritional blood parameters of patients with MMD who received revascularization at our institution from 2012 to 2021 were retrospectively reviewed. Univariate analysis and multivariable logistic regression were used to identify independent risk factors for the onset of postoperative cerebral ischemic complications. Predictive values were tested and a model incorporating these independent risk factors was created using the R program. Area under the receiver operating characteristic curve (AUC) was used for testing its discriminability.

**Results:**

Postoperative cerebral ischemic complications occurred in 32 patients of 100 included procedures. Surgery on the left hemisphere, lower admission modified Rankin Scale (mRS) score, aberrant nutritional parameters including low white blood cell (WBC), and high total cholesterol (TC) were significantly associated with cerebral ischemic complications after revascularization. The intriguing role of WBC might be explained by altered immunomodulation. The AUC of this model with novel nutritional parameters yielded a value of 0.811, presenting better predictive accuracy. Additionally, the model was visualized in the form of a nomogram and translated into a user-friendly calculator to generate individual risk.

**Conclusions:**

Surgical side, admission mRS score, WBC, and TC were independent risk factors for postoperative cerebral ischemic complications. The model composed of these four parameters was promising to be adopted in clinical practice.

## Introduction

Moyamoya disease (MMD) is an uncommon chronic intracerebral vascular disorder of unknown etiology characterized by stenosis or occlusion of the distal internal carotid artery or proximal middle cerebral artery along with a collateral network of vessels at the base of the brain (1, 2). Epidemiological data demonstrate that MMD is more prevalent in East Asian countries than the western countries (3). Even though no medical therapies were able to halt the pathologic process of moyamoya, revascularization surgeries including direct bypass, indirect bypass, and combined procedures have been proven to be effective treatments to stabilize the progression of MMD (4–6). However, postoperative complications related to compromised cerebral hemodynamics, are still a significantly impaired prognosis for patients. Intracranial hemorrhage or ischemic stroke was mostly reported adverse events due to hyper- or hypo-perfusion after revascularization (7). Of these complications, cerebral ischemia, with reported incidence ranging from 1.5 to 11.4% (8, 9) is still a challenging issue for adult patients (10) after revascularization, which impeded clinical outcome as a result of neurological deterioration and long-term neurological deficits (11). For pediatrics, the rate of perioperative cerebra infarction could be as high as 22.2% (12, 13). Therefore patients with MMD who underwent revascularization will greatly benefit from the identification of novel risk factors for cerebral ischemic complications in preoperative management.

Various clinical factors relating to the occurrence of cerebral ischemic complications post revascularization have been sporadically reported, but such data were mostly demographics like neurological status and radiological characteristics (5, 14). There is emerging literature to support the vital role of altered nutritional status in the pathogenesis of cerebral ischemic stroke (15, 16). Variables from laboratory tests and a series of nutritional screening tools are commonly used to evaluate nutritional status, but the results of nutritional screening tools may vary resulting from the complexity of computing indexes as well as expert experience of the examiners (17). Recently omics technologies are promising to identify diverse biomarkers for nutritional assessment (18), whereas they were not as easily accessed as laboratory parameters from blood tests in each medical institution. Studies were lacking in the identification of nutritional-associated risk factors in patients with MMD for the purposes of investigating the relationship with postoperative cerebral ischemic complications. Additionally, fewer data are available to generate a predictive model based on such risk factors. Taken together, we had 2 objectives in this study: (1) to explore independent risk factors for postoperative cerebral ischemic complications in terms of nutritional blood parameters from patients with MMD;(2) to create a predictive model incorporating novel risk factors for the purpose of prevention and effective management within the preoperative phase.

## Methods

### Study Design and Population

Electronic hospital files of patients with MMD who were admitted and underwent surgical revascularization at Qingdao Municipal Hospital from January 2012 to August 2021 were retrospectively screened. Patients were eligible for further analysis if (1) they were aged ≥18 and diagnosed with MMD that was confirmed by digital subtraction angiography (DSA) and/or MR angiography (MRA); (2) surgical revascularization was performed, and (3) medical records were complete. Moyamoya syndrome owing to identified causes and patients without preoperative DSA or MRA were excluded. The institutional review board was approved by the Ethnic Committee of Qingdao Municipal Hospital, and all procedures were in accordance with the guidelines of the Helsinki Declaration.

### Preoperative Evaluations

Baseline features, including age, gender, premorbid vascular risk factors, types of onset symptoms, admission modified Rankin Scale (mRS) score, surgical modalities, and radiologic profiles, were collected. Premorbid vascular risk factors covered the history of hypertension (systolic blood pressure≥ 140 mmHg and/or diastolic blood pressure ≥ 90 mmHg or use of antihypertensive drugs), history of diabetes mellitus (fasting glucose ≥7.0 mmol/L, or nonfasting glucose ≥ 11.1 mmol/L with clinical symptoms of hyperglycemia and use of hypoglycemic drugs), history of heart diseases (coronary heart disease and/or atrial fibrillation), and previous smoking status. The onset symptoms were generally categorized into two types: ischemic and hemorrhagic. The Suzuki stage determined by imaging was assessed by at least two neurosurgeons. Three surgical types, including indirect bypass, direct bypass, and combined bypass, were operated.

Body mass index (BMI), hemoglobin (Hb), serum creatinine (SCr), red cell distribution width (RDW), albumin, and prealbumin were collected as markers that are reflective of nutritional status in accordance with our previous study (19). Besides, we added blood variables regarding nutritional assessment, including white blood cell (WBC), lymphocyte count (LC,), monocyte count (MC), neutrophil count (NC), total protein (TP), sodium(Na), potassium (K), glucose(G), high-density lipoproteins cholesterol (HDL-c), low-density lipoprotein cholesterol (LDL-c), total cholesterol(TC), and triglyceride(TG), to further investigation.

For the interpretation of underlying mechanisms mediated by risk factors, prognostic nutritional index (PNI, defined as 10 × serum albumin (g/dL) + 0.005 × total lymphocyte count/mm^3^) was calculated for analysis.

### Postoperative Cerebral Ischemic Complications

Cerebral ischemic complications were categorized depending on typical symptoms that occurred within 2 weeks after surgery, and new signs of ischemia were confirmed by postoperative radiology, such as computed tomography (CT), CT perfusion, or diffusion-weighted imaging.

### Statistical Analysis

IBM SPSS Statistics 24.0 and R version 3.6.3 were used to perform all statistical analyses. Participants were categorized into two groups according to the occurrence of cerebral ischemia after revascularization. Categorical variables were presented as frequency, and the differences were compared with the chi-square test or Fisher's exact test. Following the result of Kolmogorov–Smirnov statistics, normally distributed continuous variables were presented as mean ± standard deviation and the comparisons were performed using the *t*-test, whereas nonnormally distributed continuous variables were expressed as median and interquartile range (IQRs), and intergroup differences were checked by the Mann–Whitney U-test. The variance inflation factor (VIF) and tolerance were used to check multicollinearity among variables associated with cerebral ischemic complications (*P* < 0.20) from univariate analysis and a multivariable regression model was performed. Odds ratios(OR) and 95% confidence intervals (CI) were calculated to identify potential risk factors. Pearson correlation coefficient analysis was to detect the correlation between significant risk factors and clinical indices. A predictive model was established with a receiver-operating characteristics (ROC) curve and the area under the curve (AUC) reporting the discriminatory ability. The 1,000-repetition bootstrap resampling strategy was performed for optimistic correction, and calibration was confirmed by the Hosmer–Lemeshow test. The model was visualized using R programming. DynNom and Shiny packages were exploited to create an online calculator (https://www.shinyapps.io/). All statistical tests were two-tailed, and *P* < 0.05 was considered statistically significant.

## Results

### Demographics

**Figure 1:**
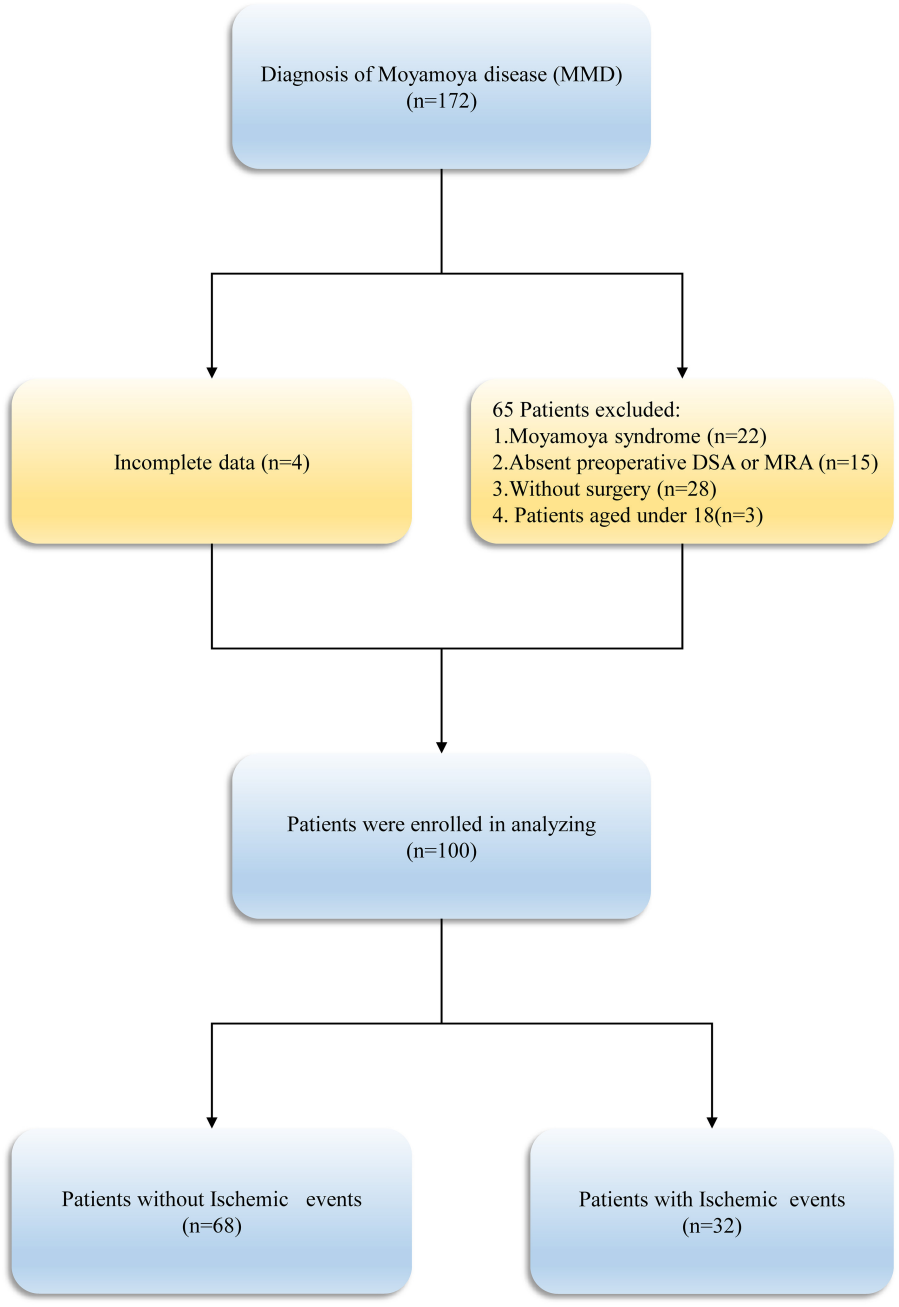
Diagram of study design.

Following the inclusion and exclusion criteria (Figure 1), a total of 100 patients with 159 hemispheres were involved in this study. Table 1 summarized the baseline participants and clinical characteristics of MMD. The patients' mean age on admission was 46.64 ± 11.42 years and men (56.0%) constituted the majority of the cohort. The clinical presentation of onset symptoms was ischemia in 82 (82.0%) patients and intracranial hemorrhage in 18(18.0%) patients. Overall, 103 revascularization surgeries were performed in 51 (51.0%) right and 49 (49.0%) in left side, with 62 (62.0%) direct bypasses, 16 (16.0%) indirect bypasses, and 22 (22.0%) combined procedures included.

**Table 1:** Baseline characteristics of the included patients.

**Characteristics**	**All Cases**
No. of patients	100
No. of hemisphere*s*^*b*^	159(100)
Age(yrs)^*a*^	46.64 ± 11.42
Se**x**^**b**^	
Male	56(56.0)
Female	44(44.0)
Premorbid histor*y*^*b*^	
Hypertension	47(47.0)
Diabetes	12(12.0)
CHD	5(5.0)
Smoker	32(32.0)
Hemisphere involve**d**^**b**^	
Bilateral	59(59.0)
Right	22(22.0)
Left	19(19.0)
Primary typ**e**^**b**^	
Ischemic	82(82.0)
Hemorrhagic	18(18.0)
Admission mR**S**^**b**^	
0–2	82(82.0)
3–6	18(18.0)
Surgical sid**e**^**b**^	
Right	51(51.0)
Left	49(49.0)
Surgery typ*e*^*b*^	
Direct bypass	62(62.0)
Indirect bypass	16(16.0)
Combined	22(22.0)
Suzuki stag**e**^**b**^	
1	1(1.0)
2	31(31.0)
3	43(43.0)
4	20(20.0)
5	5(5.0)
6	0(0)

*CHD, chronic heart disease*.

a *Mean ± SD*.

b *Percentage (%)*.

### Factors Associated With Postoperative Cerebral Ischemic Complications

**Figure 2:**
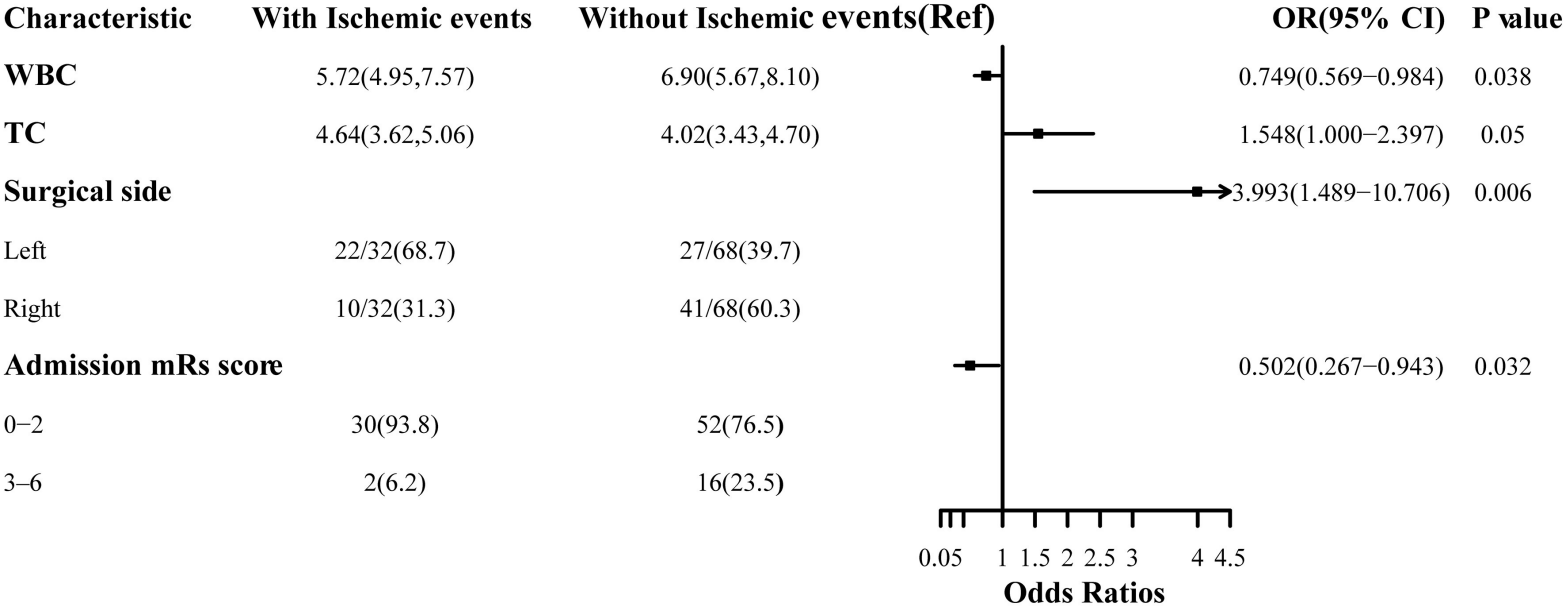
Association between risk factors and cerebral ischemic complications after revascularization in MMD.

**Figure 3:**
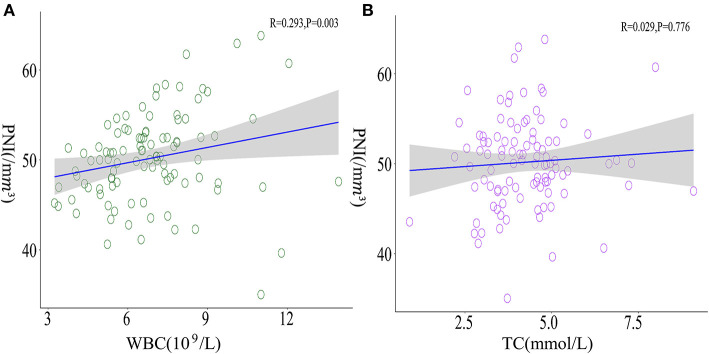
Correlations between WBC, TC, and PNI. **(A)** A positive correlation between WBC levels and PNI was observed (*r* = 0.293, *P* = 0.003) whereas **(B)** TC presented no significant correlation with PNI (*r* = 0.029, *P* = 0.776).

Table 2 exhibited newly discovered cerebral ischemic complications after revascularization surgeries. In the case group, clinical presentations covering transient ischemic attack (TIA), aphasia, epilepsy, and cerebral infarctions with confirmed radiological signs of hypoperfusion were observed in 32 (32.0%) adult patients during the rehabilitation period, among which 18 occurred after direct bypass surgery, 7 occurred after indirect bypass, and 7 occurred after combined surgery, respectively. The other 68 patients without postoperative complications were selected as the reference group. Binomial logistic regression analysis was used to explore the association between the presence of postoperative cerebral ischemic complications and clinical variables. According to univariate analysis, sex (*P* = 0.035), surgical side (*P* = 0.007), WBC (*P* = 0.021), NC (*P* = 0.014), and TC (*P* = 0.041) were found to be related to postoperative cerebral ischemic complications. A multicollinearity check was used prior to multivariate analysis(Supplementary Table 1). We next conducted multivariate logistic regression including these variables and others that have clinical importance to the development of cerebral ischemia. Results showed surgery performed on the left hemisphere (O*R* = 3.993, 95%CI 1.489–10.706), admission mRS score (OR = 0.502, 95%CI 0.267–0.943), level of WBC(OR= 0.749, 95%CI 0.569–0.984), and TC concentration(O*R* = 1.548, 95%CI 1.000–2.397) were independently associated with cerebral ischemia after revascularization surgeries (Figure 2). The tolerance was >0.5 and the VIF was <10 for all these factors, which suggested no collinearity among these variables (Supplementary Table 1). Further, we performed a backward-stepwise selection method to identify the factors in the multivariable regression analysis. Surgical side, admission mRS score, WBC, and TC values remained correlated with the development of postoperative cerebral ischemia (Supplementary Table 2). Details about clinical characteristics of patients with MMD who suffered from postoperative cerebral ischemia are summarized in Table 3. Detected by Pearson correlation coefficient analysis, the level of WBC was positively correlated with the assessment of PNI (*r* = 0.293, *P* = 0.003) while TC had no relationship with these indices (Figures 3A,B).

**Table 2:** Comparison of clinical and laboratory nutritional characteristics of patients with MMD with postoperative ischemic complications.

**Characteristics**	**All Pts (*n =* 100)**	**postoperative ischemic complications**		***P*** **-value**
		**Present (*n =* 32)**	**Absent(*n =* 68)**	
**Age(yr** **)^*a*^**	46.64 ± 11.42	45.84 ± 13.17	47.01 ± 10.59	0.703
Se*x*^*b*^				**0.035**
quadMale	56(56.0)	13(40.6)	43(63.2)	
quadFemale	44(44.0)	19(59.4)	25(36.8)	
***Premorbid history*** **^*b*^**				
quadHypertension	47(47.0)	15(46.9)	32(47.1)	0.986
quadDiabetes	12(12.0)	5(15.6)	7(10.3)	0.446
quadCHD	5(5.0)	1(3.1)	4(5.9)	0.557
quadSmoker	32(32.0)	7(21.9)	25(36.8)	0.138
***Disease involved*** **^*b*^**				0.703
quadBilateral	59(59.0)	18(56.3)	41(60.3)	
quadRight	22(22.0)	5(15.6)	17(25)	
quadLeft	19(19.0)	9(28.1)	10(14.7)	
***Primary type*** **^*b*^**				0.673
quadIschemic	82(82.0)	27(84.4)	55(80.9)	
quadHemorrhagic	18(18.0)	5(15.6)	13(19.1)	
***Admission mRs score*** **^*b*^**				0.106
quad0–2	82(82.0)	30(93.8)	52(76.5)	
quad3–6	18(18.0)	2(6.2)	16(23.5)	
***Suzuki stage*** **^*b*^**				0.325
quad1	1(1.0)	0(0)	1(1.5)	
quad2	31(31.0)	8(25.0)	23(33.8)	
quad3	43(43.0)	15(46.9)	28(41.1)	
quad4	20(20.0)	9(28.1)	11(16.2)	
quad5	5(5.0)	0(0)	5(7.4)	
quad6	0(0)	0(0)	0(0)	
***Surgical side*** **^*b*^**				**0.007**
quadRight	51(51.0)	10(31.3)	41(60.3)	
quadLeft	49(49.0)	22(68.7)	27(39.7)	
***Surgical type*** **^*b*^**				0.478
quadDirect bypass	62(62.0)	18(56.2)	44(64.7)	
quadIndirect bypass	16(16.0)	7(21.9)	9(13.2)	
quadCombined	22(22.0)	7(21.9)	15(22.1)	
**Laboratory indices**				
quadBMI (kg/*m*^2^)^*c*^	24.33(23.13,26.98)	24.99(23.00,27.55)	24.05(23.13,26.82)	0.588
quadWBC(10^9^/L)^*c*^ 6.64(5.41,7.85)		5.72(4.95,7.57)	6.90(5.67,8.10)	**0.021**
quadNC(10^9^/L)^*c*^	3.69(2.92,4.71)	3.28(2.46,4.18)	4.10(3.22,5.05)	**0.014**
quadLC(10^9^/L)^*c*^	2.02(1.57,2.40)	1.96(1.42,2.33)	2.06(1.59,2.42)	0.391
quadMC(10^9^/L)^*c*^	0.45(0.35,0.56)	0.39(0.35,0.53)	0.47(0.35,0.62)	0.217
quadRDW(fl)^*c*^	40.35(39.30,43.28)	40.55(39.30,43.58)	40.35(39.23,43.25)	0.835
quadPLR (10^9^/L)^*a*^	248.25 ± 56.29	251.97 ± 57.31	246.50 ± 56.14	0.599
quadHb(g/L)^*c*^	145.00(128.00,151.75)	134.00(122.25,150.75)	147.00(131.00,152.00)	**0.057**
quadTP(g/L)^*a*^	67.12 ± 5.36	67.10 ± 4.84	67.13 ± 5.62	0.972
quadAlbumin (g/dL)^*a*^	4.00 ± 0.33	3.98 ± 0.22	4.01 ± 0.37	0.538
quadPrealbumin (mg/L)^*c*^	286.00(234.50,329.75)	248.50(214.25,333.25)	293.00(246.25,322.50)	0.190
quadSCr(μmol/L)^*c*^	60.36(51.48,72.68)	55.36(46.16,72.29)	61.56(53.51,73.50)	0.228
quadGlucose(mg/dl)^*c*^	93.42(85.19,104.36)	90.90(85.01,100.89)	95.22(85.73,104.94)	0.338
quadK(mmol/L)^*c*^	3.94(3.76,4.17)	3.98(3.71,4.19)	3.93(3.77,4.10)	0.408
quadNa(mmol/L)^*c*^	140.00(139.00,141.90)	140.10(138.88,141.75)	140.00(139.00,141.90)	0.496
quadTG(mmol/L)^*c*^	1.14(0.89,1.55)	1.02(0.81,1.57)	1.24(0.96,1.55)	0.129
quadTC(mmol/L)^*c*^	4.16(3.46,4.82)	4.64(3.62,5.06)	4.02(3.43,4.70)	**0.041**
quadHDL-c(mmol/L)^*a*^	1.09 ± 0.28	1.14 ± 0.27	1.06 ± 0.29	0.209
quadLDL-c(mmol/L)^*c*^	2.58(1.96,3.08)	2.76(2.12,3.28)	2.39(1.91,2.92)	0.191
quadPL*R*^*c*^	122.60(95.68,158.11)	131.72(96.68,170.09)	117.29(94.17,153.26)	0.375
quadNL*R*^*c*^	1.82(1.37,2.52)	1.66(1.26,2.25)	1.84(1.41,2.57)	0.176
quadSIR*I*^*c*^	0.81(0.58,1.30)	0.67(0.52,1.04)	0.90(0.60,1.48)	0.074
quadNPA*R*^*c*^	14.20(12.80,16.10)	13.71(12.80,15.93)	14.29(12.64,16.41)	0.464

*pts, patients; CHD, chronic heart disease; BMI, Body Mass Index; WBC, white blood cell; LC, lymphocyte count; MC, monocyte count; NC, neutrophil count; RDW, red cell distribution width; Hb, hemoglobin; TP, Total Protein; SCr, serum creatinine; FFA, free fatty acids; HDL-c, high-density lipoproteins cholesterol; LDL-c, low-density lipoprotein cholesterol; TC, total cholesterol; TG, triglyceride; PLR, platelet to lymphocyte ratio;NLR, neutrophil to lymphocyte ratio; SIRI, systemic inlammation response index; NPAR, neutrophil percentage to albumin ratio; SD, standard deviation*.

a *Mean ± SD*.

b *Percentage (%)*.

c *Median (25, 75th)*.

^†^*In this and successive tables, Suzuki stage refers to the Suzuki stage of the surgically treated side*.

*The bold values indicates p values < 0.05*.

**Table 3:** Details of patients with MMD with postoperative ischemic complications.

**ID**	**Age**	**Sex**	**Type**	**Surgical** **side**	**Surgery type**	**WBC**	**TC**	**Ischemic events**	**Discharge mRS**
1	50	F	Hemorrhage	Right	IB	5.26	7.30	Cerebral infarction	3
2	43	M	Ischemia	Right	CB	4.61	4.85	Epilepsy	2
3	66	F	Ischemia	Right	DB	9.00	3.79	TIA	2
4	40	M	Ischemia	Left	DB	5.82	4.17	Motor aphasia	1
5	36	F	Ischemia	Left	DB	6.01	6.04	Motor aphasia	2
6	21	F	Ischemia	Left	CB	5.60	2.64	Motor aphasia	1
7	36	F	Ischemia	Left	DB	5.36	2.83	TIA/Motor aphasia	3
8	31	F	Ischemia	Left	DB	6.46	4.73	Anomic aphasia	4
9	62	M	Ischemia	Left	DB	8.69	3.78	Epilepsy	2
10	54	F	Ischemia	Left	CB	4.94	6.65	Transient aphasia	1
11	45	F	Ischemia	Left	CB	9.40	4.67	TIA/Cerebral hernia	4
12	29	M	Ischemia	Left	DB	7.89	5.35	Motor aphasia	2
13	29	M	Ischemia	Left	DB	7.73	3.56	TIA/ Cerebral infarction/ Motor aphasia	2
14	56	M	Ischemia	Left	DB	8.75	5.14	TIA	1
15	47	F	Hemorrhage	Right	CB	5.40	4.56	TIA	2
16	51	F	Hemorrhage	Left	DB	3.38	4.60	TIA	1
17	29	M	Ischemia	Right	DB	7.85	3.53	TIA/ Cerebral infarction	1
18	64	F	Ischemia	Right	IB	4.94	4.90	Cerebral infarction/ Epilepsy	5
									
20	21	F	Ischemia	Left	DB	7.48	4.91	TIA	2
21	57	F	Ischemia	Left	CB	5.38	4.82	Motor aphasia	2
22	69	F	Ischemia	Left	IB	7.08	5.09	Motor aphasia	2
23	47	F	Ischemia	Left	CB	5.23	6.50	Cerebral infarction/ Epilepsy	4
24	31	F	Hemorrhage	Right	DB	4.37	3.96	Epilepsy	2
25	51	M	Ischemia	Right	DB	6.50	3.17	Motor aphasia	2
26	56	F	Ischemia	Right	DB	6.83	3.90	Motor aphasia/Dysphagia	3
27	46	M	Ischemia	Left	DB	4.08	3.05	Motor aphasia	2
28	38	M	Ischemia	Left	DB	4.96	4.09	Motor aphasia	2
29	60	F	Ischemia	Left	IB	5.62	3.43	Cerebral infarction/ Epilepsy	4
30	50	F	Ischemia	Left	IB	7.60	4.87	Epilepsy	5
31	51	M	Ischemia	Left	DB	6.68	2.92	Motor aphasia	1
32	39	M	Ischemia	Left	IB	4.02	5.32	TIA/ Epilepsy	3
33	62	M	Hemorrhage	Right	CB	3.24	4.98	Epilepsy/Cerebral hernia	5

*IB, Indirect bypass; DB, Direct bypass; CB, Combined bypass; WBC, white blood cell; TC, total cholesterol; TIA, Transient ischemic attack*.

### Development of a Predictive Model for Postoperative Cerebral Ischemic Complications

**Figure 4:**
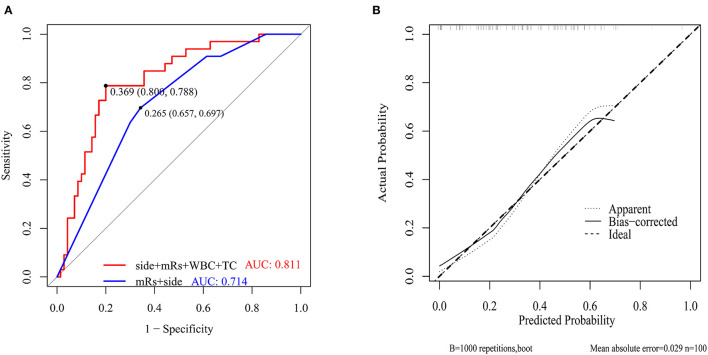
Discriminability and calibration curves for predictive model. **(A)** Discriminability between the newly established model and combination of clinical features was compared with ROC analysis. The cut-off value of our model and traditional measurements for predicting was 36.9%(sensitivity 80.0%, specificity 78.8%) and 26.5%(sensitivity 65.7%, specificity 69.7%), respectively. AUC of the prediction model showed a significant enhancement in contrast to measurements only composed of surgical side and admission mRS (0.811 vs. 0.714, *P* = 0.013 by Delong's test). **(B)** Calibration curve presented prediction of cerebral ischemic complications after revascularization between the prediction model and actual observation. The Hosmer-Lemeshow test indicated a good prediction of the nomogram (*P* = 0.692).

**Figure 5:**
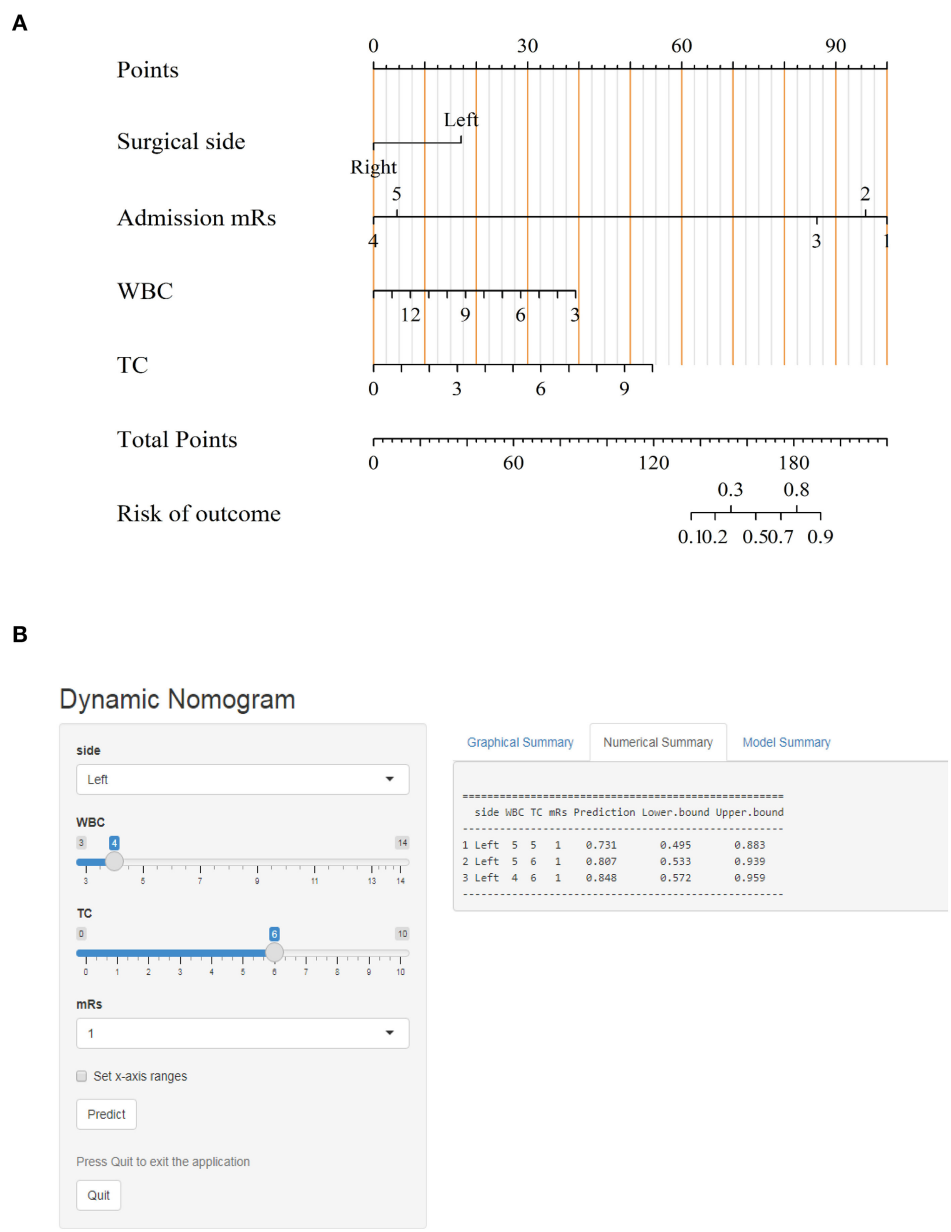
Visualization for predictive model. **(A)** Nomogram for predicting cerebral ischemic complications after revascularization in MMD. **(B)** The online calculator translated from the nomogram for generating risk of cerebral ischemic complications.

The ROC curves with regard to predictive ability of these independent risk factors for occurrence of postoperative cerebral ischemic complications are constructed in Figure 4. In contrast to the utility of surgical side and admission mRS (AUC:0.714, 95%CI: 0.615–0.8131), the inclusion of preoperative WBC and TC levels yielded to a significant improvement in predictive value (AUC:0.811, 95%CI: 0.723–0.899, DeLong's test *P* = 0.013) (Figure 4A). A calibration plot comparing the prediction of postoperative cerebral ischemic complications between the model and actual observation was created with the result of the Hosmer–Lemeshow test (*P* = 0.692), indicating good predictive accuracy (Figure 4B). Further, we not only established a nomogram incorporating these independent variables (Figure 5A), but translated it to a dynamic online calculator (https://liluo-qdmh.shinyapps.io/DynNomapp/) aiming at promoting the clinical utility of our findings (Figure 5B).

## Discussion

The present study investigated the association between clinical features, preoperative nutritional blood parameters, and the onset of cerebral ischemic complications after revascularization surgeries in patients with MMD. Our findings elicited patients with lower mRS score on initial admission, and surgeries performed on the left hemisphere were likely to encounter postoperative cerebral ischemia. Besides, nutritional blood parameters, lower WBC and higher TC levels, were identified as novel risk factors linked to the occurrence. Combined use of these four variables presented a good predictive ability for the onset of cerebral ischemic complications in MMD. To our knowledge, surgical side, WBC, and TC were newly reported independent indicators in comparison with previous research (12, 14, 20).

Revascularization is deemed as the most successful therapy to reduce the risk of stroke recurrence for patients with MMD by augmenting cerebral blood flow, but clinical outcomes remain to be challenged due to postoperative complications that are associated with impaired hemodynamic status (21). It has been discovered that patients with MMD suffer a relatively higher risk of additional ischemic events during the perioperative period (1). The frequency of TIA as a reversible symptom after revascularization surgeries in our study was similar to a rate of 6.1 % from a previous report (22), and there are still certain cases presented with TIA as the initial symptom and subsequently culminated in severe cerebral infarction or even cerebral hernia, which inevitably led to a worse outcome. Thus, it is reasonable that risk factors should be fully analyzed in preoperative management to prevent ischemic complications.

Numerous factors indicating the risk of complications after revascularization of MMD have been explored, and most are baseline characteristics like age, blood pressure, mRS on admission, or Suzuki stage (23, 24). Recently published researches have introduced several cerebral flow measurements in MMD to monitor hemodynamic status for perioperative evaluation (25, 26). Nevertheless, the requirement of expert knowledge may limit the operation of such technological advancements, and hence parameters that can be easily obtained to assess the risk of complications in patients with MMD who received revascularization are clinically needed. Complete blood count and biochemical analysis are routinely performed on each patient within initial admission and many contents are reflective of the valuable details about a patient's nutritional status (27). The association between aberrant nutrition and ischemia has been much discussed in both cardio- (28) and cerebrovascular diseases (29). Pathogenesis induced by altered nutritional status, including inflammation (30), oxidative stress (31), or lipid oxidation (32), generally contributes to the progression of vascular-ischemic events. Masahito et al. suggested that patients with MMD with preoperative malnutrition are prone to postoperative vascular complications *via* a possible vascular permeability impairment and increased inflammatory response (33). Based on exploration of nutritional blood parameters from complete blood count and biochemical analysis, we observed that patients developing postoperative cerebral ischemia had relatively lower WBC and higher TC levels before surgery.

A growing body of evidence from clinical and preclinical studies is in favor of the negative impacts of high TC level on vasculopathy, which highlights the risk of ischemic stroke (34, 35). Similar to previous studies, we assumed that inflammatory response was the underlying pathway triggered by excessive TC to provoke postoperative cerebral ischemia in patients with MMD, whereas the finding of WBC was intriguing because cerebral ischemic events are mostly reported to relate to elevated WBC level (36, 37). WBC, apart from a reliable biomarker for inflammation, was also measured as a possible indicator for the effects of immunonutrition (38, 39). In the case of such an interpretation, we took several composite indices into analysis and found that the level of WBC was positively correlated to PNI, an appliable biomarker quantifying both the nutritional and immunological status (40). The clinical values of PNI were generally explained by the two components of serum albumin concentration and total lymphocyte count in the peripheral blood (41). Low PNI might indicate lower cellular immune system activity that cannot function properly to decrease the patients' vulnerability to pathological noxae (42, 43). Since aberrant immune response can contribute to the pathogenesis of ischemic stroke (44), we hypothesized that patients with MMD with a relatively low WBC level had a relationship with aberrant immunomodulation around vessel walls, leading to altered vascular activity or disruption of the blood supply. This being the case, preoperative nutritional interventions to increase WBC as well as lower TC concentration are supposed to reduce the risk of cerebral ischemia after surgical revascularization.

Interestingly, the association between mRS on initial admission and cerebral ischemic complications after surgeries was contradictory to the clinical experience that patients with higher mRS scores are susceptible to an increased risk for complications. This finding was consistent with Li et al.'s research that admission mRS score was an independent protective factor (45). For such patients, new perfusion compensation might have been established due to experienced cerebral ischemic or hemorrhagic events, thus resulting in a relatively stable status. Hence, it is worthy to be noted that compared to patients with high mRS, perioperative managements should be carefully implemented on those who exhibited lower mRS score.

Notable strengths in our study were as follows:(1) in terms of nutritional parameters from routine blood tests, we determined that lower WBC and higher TC levels are independently associated with postoperative cerebral ischemic complications in patients with MMD who underwent revascularization. They are more practical, less expensive, and time-efficient biomarkers for clinical utility. Moreover, a predictive model incorporating these factors alongside with conventional baseline features was established in the form of nomogram, which could guide decisions on individualized and correct therapeutic strategies before surgery. Adding WBC and TC to model only covering the surgical side and mRS on admission showed a significant improvement in predicting the onset of postoperative cerebral ischemia; (2) to promote easy application in clinical practice, we translated this nomogram into a web-based calculator that may be well-adapted to a mobile device with feasible browsing. However, several limitations should be recognized:(1) this is a retrospective investigation based on single-centered data and hence potential bias may exist; (2) although the discriminative value of nomogram was good, the relatively small number of participants due to the merit of rarity and regional distribution of MMD was not easy to set up an external validation; (3) a variety of clinical factors were recorded in previously published reports, but they were not regularly measured in our preoperative measurement, which might compromise the statistical validity of our analysis. Prospective and multicenter trials are strongly recommended to support robust evidence to our findings.

## Conclusions

The preoperative nutritional status of patients with MMD is closely linked to the onset of postoperative ischemic complications. High concentration of TC and low WBC, as novel nutritional blood parameters, are independent risk factors for cerebral ischemic complications following surgical revascularization. The utility of nomogram, composed of surgical side, mRS on admission, WBC, and TC levels in preoperative management, is expected to effectively identify patients at high risk of developing cerebral ischemic complications and guide clinicians to implement appropriate nutritional interventions for prevention.

## Data Availability Statement

The original contributions presented in the study are included in the article/Supplementary Material, further inquiries can be directed to the corresponding author/s.

## Ethics Statement

The studies involving human participants were reviewed and approved by Ethnic Committee of Qingdao Municipal Hospital. Written informed consent for participation was not provided by the participants' legal guardians/next of kin because: this is a retrospective study and all data were anonymous. Written informed consent was not obtained from the individual(s), nor the minor(s)' legal guardian/next of kin, for the publication of any potentially identifiable images or data included in this article.

## Author Contributions

LL and TL were responsible for conceptualizing this study, design, data interpretation, and critical revision of the manuscript. FL and CL were joint contributors who participated in revascularization surgeries and played a major role in acquisition of clinical records, data analyzing, figures, and manuscript drafting. YS, YB, WJ, ZS, YW, ML, and WW participated in acquisition and interpretation of data. All authors read and approved the final manuscript.

## Funding

This work was supported by grants from the National Natural Science Foundation of China (82001253 to TL and 82001184 to ML).

## Conflict of Interest

The authors declare that the research was conducted in the absence of any commercial or financial relationships that could be construed as a potential conflict of interest.

## Publisher's Note

All claims expressed in this article are solely those of the authors and do not necessarily represent those of their affiliated organizations, or those of the publisher, the editors and the reviewers. Any product that may be evaluated in this article, or claim that may be made by its manufacturer, is not guaranteed or endorsed by the publisher.
