# Supplementary material for: Cerebral Ischemic Complications After Surgical Revascularization for Moyamoya Disease: Risk Factors and Development of a Predictive Model Based on Preoperative Nutritional Blood Parameters
Source: Front Nutr. 2022 Mar 10;9:842838. doi: 10.3389/fnut.2022.842838 (PMC8960452; doi:10.3389/fnut.2022.842838)
Supplement: Supplementary file 1 [file Data_Sheet_1.docx]

| **Table S1. Multicollinearity check prior to multivariate regression model**   \|  \| \| \| \| --- \| --- \| --- \| \| Factors \| Collinearity statistics \| \| \|  \| Tolerance \| VIF \| \| Sex \| .363 \| 2.756 \| \| Smoker \| .582 \| 1.717 \| \| Surgical side \| .897 \| 1.115 \| \| Admission mRs score \| .801 \| 1.249 \| \| WBC \| .072 \| 13.834 \| \| NC \| .046 \| 21.561 \| \| HGB \| .473 \| 2.113 \| \| PAB \| .536 \| 1.866 \| \| SCr \| .677 \| 1.476 \| \| TG \| .500 \| 2.000 \| \| TC \| .766 \| 1.305 \| \| HDL \| .636 \| 1.572 \| \| LDL \| .519 \| 1.927 \| \|  \|  \|  \|   **Table S2. The tolerance and VIF for significant risk factors.** | | | | |
| --- | --- | --- | --- | --- | --- | --- | --- | --- | --- | --- | --- | --- | --- | --- | --- | --- | --- | --- | --- | --- | --- | --- | --- | --- | --- | --- | --- | --- | --- | --- | --- | --- | --- | --- | --- | --- | --- | --- | --- | --- | --- | --- | --- | --- | --- | --- | --- | --- | --- | --- | --- | --- | --- | --- | --- |
| Factors | Collinearity statistics | | | |
|  | Tolerance | | VIF | |
| Surgical side | 0.986  0.938  0.948  0.972 | | 1.015 | |
| Admission mRs score |  |  | 1.066 | |
| WBC |  |  | 1.055 | |
| TC |  |  | 1.029 | |
|  |  |  |  |  |

**Table S3. Backward-stepwise multivariate logistic regression analysis of risk factors associated with postoperative ischemia.**

| **Risk Factors** | **B** | **Standard error** | **Wald** | **P value** | **OR** | **95%CI of OR** |
| --- | --- | --- | --- | --- | --- | --- |
| Surgical side | 1.384 | 0.503 | 7.569 | 0.006 | 3.993 | 1.489-10.706 |
| Admission mRs score | -0.689 | 0.322 | 4.589 | 0.032 | 0.502 | 0.267-0.943 |
| WBC | -0.290 | 0.140 | 4.298 | 0.038 | 0.749 | 0.569-0.984 |
| TC | 0.437 | 0.223 | 3.884 | 0.050 | 1.548 | 1.000-2.397 |
| Constant | -1.672 | 1.525 | 1.202 | 0.273 | 0.188 |  |
| Abbreviations: WBC=white blood cell; TC=total cholesterol; B= regression coefficient; OR=odd ratio; CI= confidence interval    **Table S4. Comparison of ROC curves**   \| New model versus Traditional model \| \| \| --- \| --- \| \| Difference between areas  Standard Error  95% CI  Z statistic  Significance level \| 0.097  0.0204 to 0.1740  2.481  0.0131 \|   Pairwise comparion of ROC curves（Delong’s test）  New model=Surgical side+ Admission mRs score+ WBC+ TC  Traditional model=Surgical side+ Admission mRs score  Abbreviations: ROC= receiver operating characteristic curve; CI= confidence interval | | | | | | |
